# Maternal Dietary Patterns, Socioeconomic Conditions, and Birth Outcomes in the MAMI-MED and Piccolipiù Italian Birth Cohorts

**DOI:** 10.3390/nu18071065

**Published:** 2026-03-26

**Authors:** Giuliana Favara, Letizia Leccese, Martina Barchitta, Francesca Candelora, Martina Culasso, Carla Ettore, Giuseppe Ettore, Luigi Gagliardi, Fabiola Galvani, Vieri Lastrucci, Claudia La Mastra, Maria Clara La Rosa, Roberta Magnano San Lio, Andrea Maugeri, Paola Pani, Lorenza Nisticò, Sonia Brescianini, Antonella Agodi

**Affiliations:** 1 Department of Medical and Surgical Sciences and Advanced Technologies “GF Ingrassia”, University of Catania, 95123 Catania, Italy; giuliana.favara@unict.it (G.F.); martina.barchitta@unict.it (M.B.); claudia.lamastra@outlook.it (C.L.M.); mariclalarosa@gmail.com (M.C.L.R.); robertamagnanosanlio@unict.it (R.M.S.L.); andrea.maugeri@unict.it (A.M.); 2 Centre for Behavioural Science and Mental Health, Istituto Superiore di Sanità, 00161 Rome, Italy; letizia.leccese@iss.it (L.L.); lorenza.nistico@iss.it (L.N.); sonia.brescianini@iss.it (S.B.); 3 Cancer Epidemiology Unit, Department of Medical Sciences and CPO-Piemonte, University of Turin, 10124 Turin, Italy; francesca.candelora@unito.it; 4 Department of Epidemiology, ASL Roma 1, Lazio Regional Health Service, 00154 Rome, Italy; m.culasso@deplazio.it; 5 Department of Obstetrics and Gynaecology, Azienda di Rilievo Nazionale e di Alta Specializzazione (ARNAS) Garibaldi Nesima, 95124 Catania, Italy; carla.ettore@hotmail.it (C.E.); giuseppe.ettore@gmail.com (G.E.); fabiolagalvani38@gmail.com (F.G.); 6 Department of Mother and Child Health, Azienda USL Toscana Nord Ovest, 56121 Pisa, Italy; l.gagliardi@usl12.toscana.it; 7 Epidemiology Unit, Meyer Children’s Hospital IRCCS, 50139 Florence, Italy; vieri.lastrucci@meyer.it; 8 Clinical Epidemiology and Public Health Research Unit, Institute for Maternal and Child Health—IRCCS “Burlo Garofolo”, 34137 Trieste, Italy; paola.pani@burlo.trieste.it

**Keywords:** dietary patterns, perinatal outcomes, birth cohorts, socioeconomic determinants

## Abstract

**Background/Objectives:** Maternal diet during pregnancy may influence neonatal outcomes, but dietary behaviours are socially patterned and were measured differently across cohorts. We therefore evaluated whether cohort-specific, partially harmonized maternal dietary patterns were associated with adverse birth outcomes after accounting for maternal and socioeconomic characteristics in two Italian birth cohorts. **Methods:** We analyzed 3234 mother–infant dyads from Piccolipiù (2011–2015) and 1564 from MAMI-MED (2020–ongoing). Maternal diet was captured by cohort-specific food questionnaires and grouped into food categories. Principal component analysis identified dietary patterns; pattern scores were categorized into tertiles and combined into five joint-adherence profiles. Logistic regression estimated odds ratios (OR) for preterm birth (PTB, <37 weeks), low birth weight (LBW, ≤2500 g), macrosomia (≥4000 g), and small/large for gestational age (SGA/LGA), with progressive adjustment for maternal age, pre-pregnancy body mass index (BMI), education, employment, and (Piccolipiù) income. **Results:** Two comparable patterns emerged in both cohorts: Western (processed foods, fried items, snacks/sweets, sugar-sweetened beverages) and Prudent (fruit, vegetables, fish, whole grains/yogurt). Western adherence was more common among younger women and those with disadvantage, whereas Prudent adherence tracked higher education, employment and income. After full adjustment, dietary profiles were not consistently associated with PTB, SGA or LGA in either cohort. In Piccolipiù, preferential Prudent adherence was associated with lower odds of LBW (OR 0.49; 95% CI 0.24–0.92) and higher odds of macrosomia (OR 1.56; 95% CI 1.06–2.30). Across cohorts, higher pre-pregnancy BMI predicted macrosomia/LGA, while lower education increased the probability of PTB and LBW. **Conclusions:** Across two Italian birth cohorts, maternal dietary patterns were socially stratified, whereas pre-pregnancy BMI and maternal education were more consistently associated with birth outcomes than dietary-pattern adherence per se.

## 1. Introduction

Within the broader framework of the Developmental Origins of Health and Disease (DOHaD), maternal nutrition is a modifiable exposure with implications for pregnancy and neonatal health. The DOHaD paradigm posits that maternal conditions during critical periods of fetal development can shape long-term health trajectories [1,2]. Because foods are consumed in combination, dietary pattern analysis, rather than single nutrients or foods, captures the multidimensional nature of maternal habitual intake and has become crucial in perinatal epidemiology [3]. For these reasons, the choice of data-driven approaches is crucial to the valid characterization of maternal dietary profiles [4]. A priori indices (e.g., diet-quality scores), a posteriori approaches (e.g., principal components analysis, cluster analysis), and hybrid approaches address complementary questions and differ with respect to assumptions, reproducibility, and interpretability [5,6,7].

Moreover, evidence indicates that higher adherence to healthy patterns during pregnancy is associated with a lower risk of preterm birth (PTB) and favorable birth size. Consistently, systematic reviews and meta-analyses report inverse associations with PTB and reduced risk of small-for-gestational-age (SGA) for maternal dietary healthier patterns, whereas unhealthy patterns are associated with lower birth weight and a higher risk of PTB [8,9,10].

Maternal dietary habits, on the other hand, are influenced by multiple sociodemographic factors—including educational level, income, employment, and working conditions—with consistent gradients documented across diverse settings [11,12,13].

Individuals with higher household incomes generally have access to a broader variety of nutritious foods, whereas constrained resources are associated with reliance on cheaper, energy-dense options that are poor in essential nutrients [14,15,16].

Educational attainment influences food choice and nutrition literacy. In fact, higher education is associated with more balanced diets and better understanding of the role of key nutrients, while lower education may hinder the adoption of healthful patterns [17]. Finally, working conditions shape both income and the time available for meal preparation. Irregular schedules and high job-related stress are frequently linked to greater consumption of fast foods or ready-made meals, with consequent penalization of fresh and nutrient-dense foods [18].

Several studies have also shown that socioeconomic factors and other maternal characteristics are associated with reproductive outcomes. Shorter maternal education, in particular, has been associated with a higher risk of a preterm delivery across 12 European birth cohorts [19].

However, two issues remain insufficiently addressed in the literature. First, maternal diet is strongly socially patterned, and it is therefore unclear whether associations between dietary patterns and perinatal outcomes persist after accounting for maternal socioeconomic conditions and pre-pregnancy BMI. Second, few studies have examined whether broadly similar findings emerge across cohorts recruited in different periods and regions and assessed with different dietary instruments. In the present study, we performed parallel analyses in two Italian birth cohorts with partially harmonized dietary exposures to (i) derive cohort-specific dietary patterns, (ii) describe their sociodemographic correlates, and (iii) assess whether adherence to these patterns is associated with birth outcomes after sequential adjustment for maternal and socioeconomic characteristics. We approached cross-cohort comparison as an assessment of consistency in broad patterns of association rather than as a direct replication of identical exposures.

## 2. Materials and Methods

### 2.1. Study Population and Design

The Piccolipiù birth cohort is an Italian multicenter study designed to evaluate the impact of prenatal and postnatal exposures on later health. The cohort comprises 3358 children born between 2011 and 2015 in six hospitals of five Italian cities—Turin, Trieste, Florence, Viareggio, and Rome. The Piccolipiù study protocol was approved by the Ethics Committee of the Local Health Unit Roma E, the national coordinating center (Prot. CE/82, 9 June 2011), by the ethics committees of each local center [20], and is in line with the Declaration of Helsinki. Eligible participants were mothers aged ≥18 years who resided in one of the participating municipalities and planned to deliver in a study hospital. Women were contacted during pregnancy or at delivery and were asked to complete a baseline questionnaire, with questions on demographics, environmental exposures, and mother’s health. Additional information was obtained either from medical records or directly from the mother within the 48 h after delivery. Participants were then contacted at 6, 12, 24 months, 4 and 6 years after delivery and asked to fill in self-administered questionnaires. All parents provided written informed consent at baseline and at each subsequent follow-up.

The MAMI-MED cohort is a prospective study initiated in December 2020, comprises 1564 pregnant women, and aims to evaluate the influence of social, environmental, behavioral, and molecular factors on the health of mother–child dyads. The study protocol aligns with the methodology of the “Mamma and Bambino” cohort, which has been ongoing in Catania since 2015 [21].

Participants were recruited among pregnant women attending their first-trimester prenatal visit as part of routine care at the Azienda di Rilievo Nazionale e di Alta Specializzazione (ARNAS) Garibaldi Nesima Hospital in Catania, Italy. Women were included if they attended their first prenatal visit within the first trimester of pregnancy and expressed their intention to give birth at the above-mentioned operative unit.

The study plan includes interviews at recruitment, at delivery, and after 12, 24, and 48 months to collect information regarding the health, socioeconomic status, and lifestyles of the parents, as well as data concerning health, habits, and diet of the child.

The MAMI-MED study protocol was originally approved by the Catania 2 Ethics Committee before recruitment started in December 2020 (initial approval: Protocol 71/2020/CECT2). The protocol numbers 487/CE, 157/CEL, and 206/CEL refer to subsequent amendments and/or renewals. All women were fully informed about the study purpose and procedures and provided written informed consent prior to their inclusion.

### 2.2. Data Collection

The variables considered are described in detail in Table 1 that also displays similarities and differences between cohort variables.

Variables analyzed included parental, sociodemographic, and maternal–child factors, namely: maternal age at delivery, pre-pregnancy body mass index (BMI) and BMI category (underweight, normal weight, overweight, obesity), education level, employment status, recruitment center, equivalized household income indicator (EHII), only in Piccolipiù, smoking status, parity (nulliparous vs. multiparous), gestational age at birth, birth weight and length, mode of delivery (vaginal vs. caesarean), and obstetric history. Pre-gestational BMI, defined as weight in kilograms divided by height in squared meters, was classified according to World Health Organization (WHO) criteria as underweight (<18.5), normal weight (18.5–24.9), overweight (25–29.9), and obese (≥30) [22].

Neonatal outcomes of interest included preterm birth (PTB, defined as spontaneous delivery before 37 weeks of gestation), low birth weight (LBW), macrosomia, and birth weight for gestational age, classified as small (SGA), adequate (AGA), or large for gestational age (LGA) based on sex-specific national reference charts [23].

Analyses were conducted on mother–child dyads with complete data available through delivery follow-up. Information on pregnancy, delivery, and neonatal outcomes was collected at birth using ad hoc questionnaires.

### 2.3. Maternal Dietary Data

In the Piccolipiù cohort, maternal diet information was obtained through a structured food questionnaire self-administered close to delivery. Although the questionnaire has not been formally validated, it was included as part of the multipurpose design of this study to capture general dietary patterns rather than to provide precise quantitative intake estimates. Mothers were asked to indicate their frequency of consumption for 21 food groups—pasta, red meat, white meat, processed meats, fish, seafood, eggs, legumes, potatoes, raw vegetables, cooked vegetables, fruit, cheese, mayonnaise, canned products, fried foods, sweets, snacks, yogurt, milk, soft drinks, classified in six categories (never; <1 time/week; 1–2 times/week; 3–5 times/week; 6–7 times/week; >1 time/day). Moreover, three more food groups included non-decaffeinated coffee, non-decaffeinated tea, and cola for which women were also asked the average number of cups consumed in each trimester during pregnancy.

The MAMI-MED birth cohort collected maternal dietary information at recruitment using a 95-item semi-quantitative food frequency questionnaire (FFQ) administered by trained epidemiologists to capture intake in the month preceding enrollment.

Women were asked to indicate their frequency of consumption, which was classified into 12 categories (“Never”, “1 to 3 times per month”, “1 to 6 times per week”, “Once per day”, “Twice or more per day”), and the serving size, categorized as small, medium, or large. The medium serving size was defined using standard weight or volume measures commonly consumed in the Italian population, while small and large serving sizes were defined as half or 1.5 times (or more) a medium serving size, respectively. An illustrative photograph atlas was provided to help estimate the quantity of each food item and minimize inaccuracies. For each food group, daily intakes were calculated by multiplying the frequency of consumption by the portion size, by using the U.S. Department of Agriculture (USDA) Food Composition Database https://fdc.nal.usda.gov/ adapted to typical Italian foods [24].

Because the two cohorts differed substantially in questionnaire structure, level of detail, timing of administration, and validation status, dietary data were analyzed separately within each cohort and then interpreted comparatively only at the level of broad dietary dimensions. Accordingly, the cross-cohort comparison should be understood as a comparison of partially harmonized exposure constructs rather than as a direct comparison of identical dietary measures.

### 2.4. Statistical Analyses

#### 2.4.1. Principal Component Analysis for Dietary Pattern Identification

Food-intake variables were z-standardized (mean = 0, SD = 1) to ensure comparability across food groups. In the Piccolipiù cohort, participants with missing dietary data (n = 104; ~3%) were excluded, leaving 3234 mothers, and PCA was conducted on 24 predefined food groups. For the MAMI-MED cohort, the 95 FFQ items were aggregated into 39 food groups (retaining some items as standalone, e.g., pizza and coffee, and collapsing broader categories such as fruit and vegetables). In both cohorts, maternal dietary patterns were identified using Principal Component Analysis (PCA) applied to standardized food-group frequencies. Importantly, all food groups and their corresponding loadings were retained in the PCA solution and in the computation of individual factor scores; the cut-offs were applied only to describe and name the patterns (i.e., to highlight the food groups most strongly characterizing each component) and did not affect downstream analyses.

Because the two cohorts differed in questionnaire length, level of food aggregation, and timing of dietary assessment, PCA was conducted separately within each cohort. We retained two components because they were the only components that simultaneously satisfied the prespecified criteria of eigenvalue ≥2, visual inflection of the scree plot, and nutritional interpretability. Additional components were explored but were less stable and added limited interpretive value. Varimax rotation was chosen to maximize separation between food groups and improve the readability of the retained dimensions. The loading cut-offs were used exclusively as descriptive aids to label the components and were not used to compute factor scores or define exposure categories. The higher descriptive threshold in Piccolipiù (≥0.40) and the more inclusive threshold in MAMI-MED (≥0.20) reflected differences in food-group aggregation and reporting heterogeneity; therefore, cross-cohort comparisons were based on overall pattern composition rather than on one-to-one correspondence of individual loadings.

The two retained components explained approximately 21% cumulative variance in Piccolipiù, and 16% in MAMI-MED. Although the cumulative variance explained by the first two components appears modest, this is consistent with the range typically observed in nutritional epidemiology where PCA is applied to derive dietary patterns. In this context, PCA is intended to identify interpretable dimensions of habitual diet (e.g., Western, Prudent) rather than to capture a large proportion of total dietary variance. Previous studies applying PCA to food frequency questionnaire data have similarly reported interpretable patterns with moderate cumulative explained variance [25].

For each cohort, individual adherence (factor) scores to each dietary pattern were calculated as weighted linear combinations of standardized food intakes and rotated factor loadings. These scores reflect the degree of conformity of an individual’s diet to each identified pattern, with higher values indicating stronger adherence. These continuous scores were then categorized into tertiles to facilitate interpretation and to identify women with low, intermediate, or high adherence to each pattern (T1–T3: low–high adherence). We subsequently combined tertiles from the two dominant patterns into five joint-adherence profiles to distinguish clearly discordant adherence (high adherence to one pattern and low adherence to the other), intermediate preference, and mixed/no-clear-preference profiles: Accordingly, joint adherence to the two dominant patterns was summarized into: exclusive adherence (T3 in one pattern and T1 in the other), preferential adherence (adjacent tertiles, e.g., T1–T2 or T2–T3 combinations), and no clear preference (same tertile in both). This categorization was defined a priori as a pragmatic summary of the relative balance between the two dominant dietary dimensions.

#### 2.4.2. Comparison Among Groups of Dietary Adherences

Both cohorts were described using medians and interquartile ranges (IQRs for continuous variables, and relative frequencies for categorical variables). The assumption of normality for continuous variables was assessed using the Kolmogorov–Smirnov test. Non-normally distributed continuous maternal variables (age, pre-pregnancy BMI) and neonatal characteristics (birth weight, birth length and gestational age) were compared between dietary adherence groups using non-parametric tests (Kruskal–Wallis test for more than two groups). When significant differences were detected, post hoc pairwise comparisons were performed using the Bonferroni correction. Associations between categorical variables were evaluated using Pearson’s Chi-square test.

#### 2.4.3. Multivariable Models

Adjusted associations between maternal dietary profiles and maternal and infant outcomes were evaluated using multivariable logistic regression models within the study cohort, accounting for cohort-specific confounders. Outcomes of interest included preterm birth (PTB), low birth weight (LBW), macrosomia, and birth weight for gestational age (LGA and SGA vs. AGA).

For each outcome, three logistic regression models were fitted. Model 1 included maternal dietary adherence as the only independent variable. Model 2 was additionally adjusted for maternal age and maternal nutritional status (pre-pregnancy BMI or equivalent cohort-specific measure). Model 3 was further adjusted for socioeconomic factors, including occupational status, educational level, and, where available, household income (EHII).

Covariates for the primary models were selected a priori to prioritize variables available in harmonized form across cohorts and to separate maternal characteristics from socioeconomic indicators. Maternal age and pre-pregnancy BMI were included as core maternal characteristics associated with both dietary behaviours and fetal growth/gestational duration. Education, employment, and household income (available only in Piccolipiù) were then added to capture the socioeconomic patterning of diet and birth outcomes. Smoking during pregnancy, parity, gestational weight gain, gestational diabetes, and other obstetric variables were not included in the primary harmonized models because their availability and coding differed across cohorts and, for some variables, adjustment could introduce overcontrol if they act as intermediates rather than baseline confounders.

Regression coefficients are reported as odds ratios (ORs) with 95% confidence intervals (CIs). All statistical analyses were performed using R statistical software (R Foundation for Statistical Computing, Vienna, Austria; version 4.2.2), with RStudio used as the integrated development environment for the Piccolipiù cohort, while SPSS software, version 26.0 (SPSS Inc., Chicago, IL, USA) was used in the MAMI-MED cohort.

## 3. Results

### 3.1. Characteristics of Study Population

Characteristics of the Piccolipiù study population are reported in Table S1 in the Supplementary File. Of the 3358 mothers enrolled, information on nutrition during pregnancy was available for 3234 (96%). The median age at delivery was 34 years (IQR = 7). When stratified by pre-pregnancy BMI, 7.6% were underweight, 72.5% normal weight, 13.5% overweight, and 5.7% obese. About socioeconomic status, approximately half of the families had a high equivalized household income (50.6%, n = 1639), consistent with the high maternal education (university degree or higher) levels observed in the sample (45%, n = 1459). During pregnancy, 55% reported abstaining from alcohol consumption. The median birth weight was 3337 g, and the median gestational age was 40 weeks.

Characteristics of the women included (n = 1.564) in the MAMI-MED cohort are reported in Table S2. The median age was 31.0 years (IQR = 7.0); median weight was 63.0 kg (IQR = 17.0) and pre-pregnancy BMI 23.3 kg/m^2^ (IQR = 6.0). Regarding sociodemographic factors, 24.3% had a low educational level, 50.8% medium, and 24.9% high; 52.3% were employed at enrollment. Current smoking was reported by 9.3% of participants. Based on pre-pregnancy BMI, 6.0% were underweight, 58.3% normal weight, 22.4% overweight, and 13.2% obese. Gestational weight gain was reduced in 38.2%, adequate in 32.2%, and excessive in 29.6%. Multiparity was present in 46.4% of women.

### 3.2. Dietary Patterns Identification

**Figure 1:**
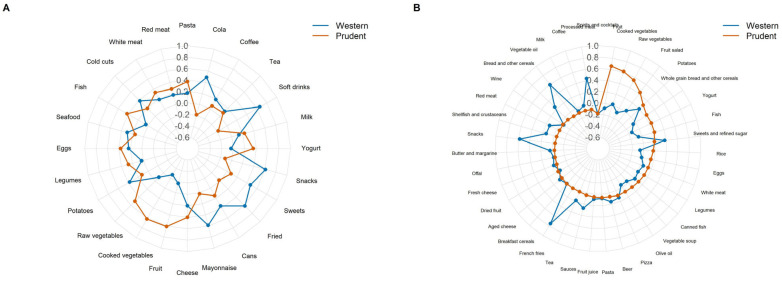
Dietary profiles of Western and Prudent patterns based on food-group loadings in Piccolipiù (**A**) and MAMI-MED cohorts (**B**).

Figure 1A presents the results of the PCA conducted in the Piccolipiù cohort with factor loadings characterizing the two main dietary patterns identified. The first pattern, labeled “Western”, was characterized by high positive loadings for soft drinks (0.66), fried foods (0.62), snacks (0.61), mayonnaise (0.59), cola (0.49), and sweets (0.47). The second pattern, labeled “Prudent”, showed strong positive loadings for cooked vegetables (0.62), fruits (0.61), raw vegetables (0.50), and fish (0.42).

Similarly, in the MAMI-MED cohort, PCA identified two predominant dietary patterns (Figure 1B): the first pattern, labeled “Western”, was characterized by high positive loadings for French fries (0.75), vegetable oils (0.59), salty snacks (0.58), processed meats (0.44), sweets and refined sugars (0.38), sauces (0.27), bread and other refined cereals (0.25), and potatoes (0.20). The second pattern, labeled “Prudent”, was defined by high loadings for fresh fruit (0.66), cooked vegetables (0.62), raw vegetables (0.55), fruit salad (0.43), potatoes (0.30), yogurt (0.23), whole-grain cereals (0.23), and fish (0.23).

### 3.3. Associations of Dietary Patterns and Maternal Characteristics

In the Piccolipiù cohort, as shown in Table 2, maternal age and BMI differed across dietary adherence groups (*p* < 0.001 and *p* = 0.011 respectively).

Exclusive or preferential adherence to the Western pattern was associated with higher BMI and greater prevalence of obesity. Bonferroni-adjusted pairwise comparisons revealed significant differences in maternal age (*p* < 0.001) for most group combinations, while for BMI, the only significant difference emerged between women exclusively adhering to the Western pattern and those exclusively adhering to the Prudent one. Regarding socioeconomic determinants, women exclusively adhering to the Western dietary pattern were more likely to fall into the low/medium-income category (57.27%, n = 197) and have medium/low education (70.6%, n = 243). In contrast, those adhering exclusively to the Prudent pattern were more often in the higher income category (67.23%, n = 238), had higher education levels (60.45%, n = 214). All associations suggested a link between socioeconomic advantage and healthier dietary choices during pregnancy (Table 2).

For MAMI-MED, when adherence was categorized into exclusive, preferential, or no preference, significant differences in maternal age were observed across the five categories (*p* < 0.001), and these differences remained significant after Bonferroni correction. Women exclusively adherent to the Prudent pattern were significantly older than those exclusively adherent to the Western pattern, preferentially adherent to the Western pattern, and those with no preference (all *p* < 0.001). Differences were also observed between women preferentially adherent to the Western and Prudent patterns (*p* < 0.001). No significant differences were detected between preferentially and exclusively Prudent women (*p* = 0.77), nor between preferentially Western women and those with no preference (*p* = 0.106). No significant differences emerged for maternal weight, pre-pregnancy BMI, or nutritional status across adherence categories. In contrast, significant differences were observed for socio-economic determinants.

Women exclusively adherent to the Western pattern were more likely to have a medium-to-low educational level (93.5%, *p* < 0.001), to be unemployed (67.6%, *p* < 0.001), and to be economically inactive (67.6%, *p* < 0.001), compared to women adhering to the Prudent pattern (Table 3).

### 3.4. Maternal and Neonatal Outcomes

Maternal and neonatal outcomes in the Piccolipiù cohort are reported in Table S3. Overall, 72.8% (n = 2356) had a natural delivery. Preterm births (<37 weeks) accounted for 4.8% (n = 154). Regarding birth weight, 2.7% (n = 87) of newborns had low birth weight, 6.4% (n = 206) were classified as macrosomic, 11% (n = 357) as SGA, and 8.6% (n = 277) as LGA. The mean gestational age at delivery was 40 weeks, with a median birth weight of 3340 g and median length of 50 cm.

Descriptive analyses of maternal and neonatal characteristics in the MAMI-MED cohort are presented in Table S4, including a total of 1564 mother–infant pairs. The median gestational age at delivery was 39 weeks (IQR = 2), and 69.1% of women delivered vaginally. With respect to neonatal outcomes, 5.9% of infants were born preterm and 6.3% had low birthweight, while the median birthweight was 3272 g (IQR = 560 g) and the median birth length was 50 cm (IQR = 2 cm). Macrosomia was observed in 3.8% of neonates, whereas the majority (95.7%) had normal birthweight. When birthweight was evaluated according to gestational age, 9.5% of newborns were classified as SGA, 79.7% as AGA, and 9.6% as LGA.

### 3.5. Multivariable Regression Models

**Figure 2:**
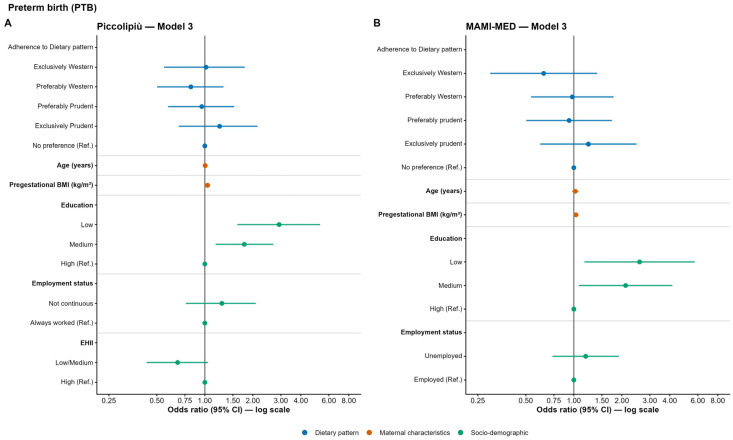
Odds ratios (OR) and 95% confidence intervals (CI) for the association between maternal adherence to dietary patterns and preterm birth in the Piccolipiù (**A**) and MAMI-MED cohort (**B**).

In the Piccolipiù cohort, no significant associations were observed for PTB in the unadjusted model (Model 1). However, after full adjustment (Model 3), women with low (OR = 2.92; 95% CI: 1.59–5.29; *p* < 0.001) and medium (OR = 1.77; 95% CI: 1.17–2.69; *p* = 0.007) education levels were more likely to experience PTB than those with high education (Figure 2A and Table S5 for full model results).

Similarly, in the MAMI-MED cohort, there was no evidence of association of the dietary pattern with PTB in the unadjusted model (Model 1). After adjusting for maternal age and pre-pregnancy BMI (Model 2), the probability of PTB was positively associated with increasing pre-pregnancy BMI (OR = 1.045; 95% CI: 1.009–1.082; *p* = 0.013).

After further adjustment for occupational status and educational level (Model 3), women with low (OR = 2.54; 95% CI: 1.15–5.63; *p* = 0.021) and medium (OR = 2.09; 95% CI: 1.06–4.11; *p* = 0.032) educational levels had a significantly higher probability of PTB compared to those with a high educational level (Figure 2B and Table S6 for full model results).

**Figure 3:**
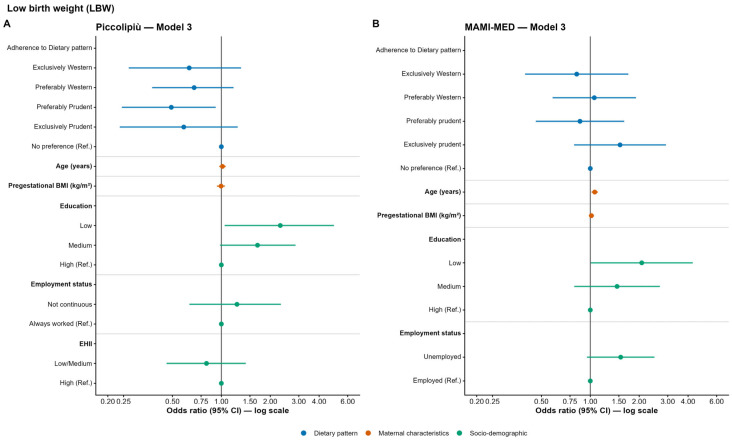
Odds ratios (OR) and 95% confidence intervals (CI) for the association between maternal adherence to dietary patterns and low birth weight in the Piccolipiù (**A**) and MAMI-MED cohort (**B**).

In the Piccolipiù cohort, women with preferential adherence to the Prudent dietary pattern showed significantly lower odds of delivering a LBW infant compared to women with no preference across all models (Model 1: OR = 0.50; 95% CI: 0.25–0.91; *p* = 0.031; Model 2: OR = 0.50; 95% CI: 0.25–0.91; *p* = 0.031; Model 3: OR = 0.49; 95% CI: 0.24–0.92; *p* = 0.035). In the fully adjusted model (Model 3), women with a low educational level also had significantly higher odds of low birth weight compared to those with a high educational level (OR = 2.31; 95% CI: 1.05–4.93; *p* = 0.034) (Figure 3A and Table S7 for full model results).

In the MAMI-MED cohort, no significant associations with LBW were observed in the unadjusted (Model 1) or partially adjusted (Model 2) models. However, in the fully adjusted model (Model 3), maternal age was significantly associated with LBW (OR = 1.065; 95% CI: 1.02–1.11; *p* = 0.006), indicating that each one-year increase in maternal age was associated with an approximately 6% higher odds of delivering a LBW infant. Additionally, women with a low educational level showed a significantly higher probability of LBW compared to those with a high educational level (OR = 2.074; 95% CI: 1.007–4.273; *p* = 0.048) (Figure 3B and Table S8 for full model results).

**Figure 4:**
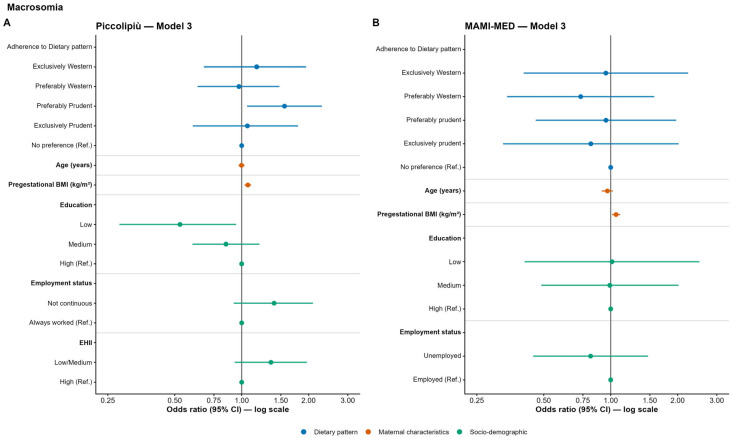
Odds ratios (OR) and 95% confidence intervals (CI) for the association between maternal adherence to dietary patterns and macrosomia in the Piccolipiù (**A**) and MAMI-MED cohort (**B**).

In Piccolipiù, women with a preferably Prudent dietary pattern showed significantly higher odds of macrosomia compared to those with no preference (Model 1: OR = 1.46; 95% CI: 1.01–2.11; *p* = 0.043; Model 2: OR = 1.52; 95% CI: 1.05–2.21; *p* = 0.027; Model 3: OR = 1.56; 95% CI: 1.06–2.30; *p* = 0.024). In all adjusted models (Model 2 and Model 3), higher pre-pregnancy BMI was also significantly associated with increased odds of macrosomia (Model 2: OR = 1.07; 95% CI: 1.04–1.10; *p* < 0.001; Model 3: OR = 1.07; 95% CI: 1.03–1.10; *p* < 0.001). Conversely, in the fully adjusted model, women with a low educational level had significantly lower odds of macrosomia compared to those with a high educational level (OR = 0.53; 95% CI: 0.28–0.94; *p* = 0.037, Figure 4A and Table S9 for full model results).

Regarding macrosomia in the MAMI-MED cohort, no significant associations with dietary patterns were observed in the unadjusted model (Model 1). In Model 2, after adjustment for maternal age, pre-pregnancy BMI was significantly associated with macrosomia, with each one-unit increase in BMI corresponding to an approximately 5% higher odds of delivering a macrosomic infant (OR = 1.054; 95% CI: 1.012–1.098; *p* = 0.012).

This association remained statistically significant in the fully adjusted model (Model 3), after further adjustment for educational level and occupational status (OR = 1.057; 95% CI: 1.014–1.103; *p* = 0.010) (Figure 4B and Table S10 for full model results).

**Figure 5:**
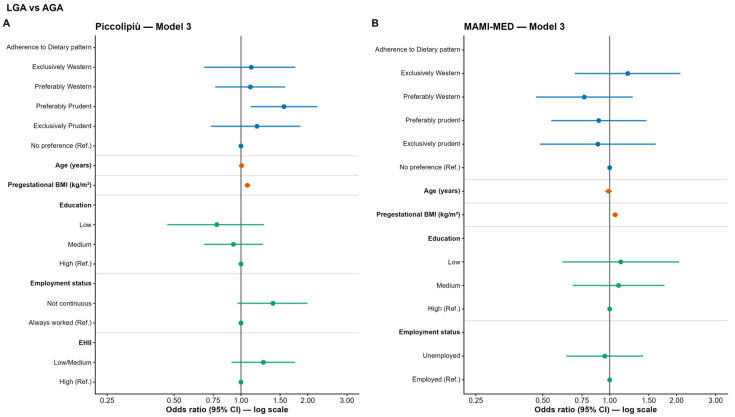
Odds ratios (OR) and 95% confidence intervals (CI) for the association between maternal adherence to dietary patterns and LGA vs. AGA in the Piccolipiù (**A**) and MAMI-MED cohort (**B**).

In the Piccolipiù cohort, women with a preferably Prudent dietary pattern showed significantly higher odds of having LGA children compared to those with no preference only in model 1 (Model 1: OR = 1.457; 95% CI: 1.04–2.03; *p* = 0.027). The association disappears in models 2 and 3. Instead, in the adjusted models (Model 2 and Model 3), higher pre-pregnancy BMI was significantly associated with increased odds of LGA (Model 3: OR = 1.07; 95% CI: 1.04–1.10; *p* < 0.001) (Figure 5A and Table S11 for full model results).

Similarly, in the MAMI-MED cohort, no significant associations between dietary patterns and LGA were observed. In Model 2, pre-pregnancy BMI was positively associated with LGA, with each one-unit increase in BMI corresponding to an approximately 6% higher odds of delivering an LGA infant (OR = 1.058; 95% CI: 1.028–1.089; *p* < 0.001). This association remained significant in the fully adjusted model (Model 3) (OR = 1.059; 95% CI: 1.028–1.091; *p* < 0.001) (Figure 5B and Table S12 for full model results).

**Figure 6:**
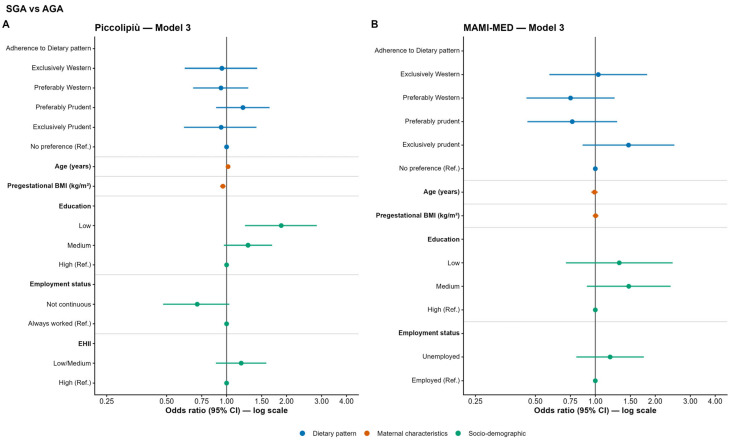
Odds ratios (OR) and 95% confidence intervals (CI) for the association between maternal adherence to dietary patterns and SGA vs. AGA in the Piccolipiù (**A**) and MAMI-MED cohort (**B**).

In the Piccolipiù cohort, no significant associations were observed between dietary pattern adherence and the likelihood of having SGA children compared to AGA. In the adjusted models (Model 2 and Model 3), higher pre-pregnancy BMI was significantly associated with decreased odds of SGA (Model 3: OR = 0.96; 95% CI: 0.93–0.99; *p* < 0.01). In the fully adjusted model, women with low educational levels had a significantly increased odds of SGA, almost double that of those with high educational attainment (OR = 1.88; 95% CI: 1.24–2.84; *p* = 0.003) (Figure 6A and Table S13 for full model results). In the MAMI-MED cohort, no significant associations with SGA were observed across all three models (Figure 6B and Table S14 for full model results).

## 4. Discussion

In the present study, two principal dietary patterns were identified across two Italian birth cohorts. Despite their derivation from women living in distinct Italian regions, a broadly comparable pair of dietary patterns was identified in each cohort. In Piccolipiù, a “Western” pattern—characterized by sugar-sweetened beverages, fried foods, snacks, sweets, and mayonnaise—and a “Prudent” pattern—rich in fish, vegetables, and fruit—were observed; in the MAMI-MED cohort, a “Western” pattern—defined by French fries, salty snacks, processed meats, and refined cereals—and a “Prudent” pattern—characterized by fruit, vegetables, whole grains, fish, and yogurt—emerged. Interestingly, in both our cohorts, adherence to less favorable patterns was more common among younger women and those with less advantaged socioeconomic conditions, while adherence to “Prudent” patterns was more frequent among women with higher education, employment, and income. These results have already been seen in other birth cohorts [13,26]. In both our cohorts, findings suggest that maternal characteristics at recruitment, rather than dietary-pattern adherence during pregnancy, mostly shape perinatal risk. Indeed, pre-pregnancy nutritional status/BMI was strongly associated with macrosomia and LGA, and lower educational levels increased the probability of preterm birth. Higher pre-pregnancy BMI was associated with an increased probability of macrosomia and LGA, indicating that each incremental increase in BMI at conception contributes to a higher risk of excessive fetal growth.

Moreover, educational attainment showed an independent association with perinatal outcomes, particularly in the Piccolipiù cohort, where lower educational levels were significantly associated with preterm birth, low birth weight, macrosomia, and SGA. This highlights maternal education as a key socioeconomic determinant of perinatal health, acting alongside pre-pregnancy BMI, with a more prominent role in Piccolipiù than in MAMI-MED.

Overall, our results align with recent reviews and meta-analyses reporting heterogeneous and, at times, inconsistent associations between maternal dietary patterns and preterm birth or neonatal size [8,9,10]. They are also broadly consistent with more recent cohort evidence showing that socially advantaged women are more likely to follow healthier pregnancy diets, whereas associations with neonatal outcomes become weaker and more context-dependent after accounting for maternal BMI and socioeconomic characteristics [27]. One plausible reason why our results differ from some previously reported protective associations is the substantial heterogeneity across studies in dietary assessment tools, timing of exposure measurement, derivation of dietary patterns, and covariate adjustment. In our study, these issues are especially relevant because the two cohorts relied on different dietary instruments and captured maternal diet at different stages of pregnancy. In Piccolipiù, moreover, preferential adherence to the prudent pattern was associated with lower odds of LBW but higher odds of macrosomia. Because these associations were confined to one cohort, did not translate into a consistent pattern across outcomes after full adjustment, and involved relatively uncommon outcomes, they should be interpreted cautiously as there might be a problem of inverse causality.

In our cohorts, a socioeconomic gradient was also observed, characterized by healthier dietary patterns among more educated and employed women.

These results are coherent with multicentre European analyses showing higher dietary quality in pregnant women with higher educational level and social stability [27,28,29]. Results obtained within the MAMI-MED and Piccolipiù cohorts further emphasized the crucial role of pre-pregnancy BMI as a determinant of macrosomia and LGA, particularly in the MAMI-MED cohort, where increasing BMI values were consistently associated with a higher probability of excessive fetal growth. This is in line with meta-analyses reporting increased risks of LGA/macrosomia among women with elevated BMI and, conversely, lower risks among underweight women compared to obese ones [30,31,32]. Moreover, mediation and interaction analyses should investigate pathways linking dietary profiles, pre-pregnancy BMI and gestational weight gain to adverse outcomes, given robust evidence for the roles of BMI and gestational weight gain in the genesis of macrosomia/LGA [33].

From a clinical and public-health perspective, our findings suggest that dietary counselling during pregnancy should not be considered in isolation from the social conditions that shape food choices. Interventions may be more effective when nutritional advice is integrated with preconception weight management and targeted support for younger and socioeconomically disadvantaged women. From a research perspective, interpreting our findings within the DOHaD framework is consistent with the hypothesis that the prenatal environment (e.g., nutritional, sociodemographic and life-style factors, air pollution) could shape offspring disease risk across the life course, potentially via epigenetic programming, modulation of low-grade inflammation, and alterations in placental function [34,35].

Our study has several limitations that should be considered. Among these, the different nature and validation status of the dietary instruments across cohorts may introduce potential exposure misclassification. Differences in the timing of dietary assessment—near delivery in Piccolipiù and during the first trimester in MAMI-MED—may have introduced behavior changes triggered by diagnosis or counselling during pregnancy. Thus, harmonization of instruments and protocols across cohorts would enhance comparability. Moreover, the lack of repeated trimester-specific dietary measures and the absence of objective nutritional biomarkers limit causal inference and expose the study to the measurement error typical of observational nutritional epidemiology. This in turn suggests that prospective studies with repeated trimester-specific dietary assessments, integrated with multiple biomarkers and digital dietary-assessment tools, could reduce misclassification bias and strengthen causal inference. We have also made some stringent choices in terms of number of components retained and thresholds for factor loadings. While these choices were made to enhance interpretability of the derived dietary patterns, they may have limited the ability to capture the full complexity and heterogeneity of dietary behaviors, potentially resulting in fewer or less distinct dietary patterns being identified. Finally, statistical power for relatively rare outcomes may be insufficient to detect modest associations, and generalizability is restricted to Italian hospital-based contexts with specific socio-demographic characteristics.

In addition, the present work has some strengths to be considered. Firstly, the study is based on two independent Italian birth cohorts recruited in different regions and periods, which enabled a parallel assessment of whether broad patterns of association were consistent across distinct contexts. At the same time, because dietary exposures were only partially harmonized across cohorts, this design should not be interpreted as a strict replication study but rather as a comparison of conceptually similar dietary dimensions measured with different tools. Secondly, dietary patterns were identified using data-driven methods and adherence categories are tertile-based, facilitating comparability of dietary behaviors across cohorts. Third, rigorous confounder control was employed: multivariable adjustment for maternal age, pre-pregnancy BMI, educational level, employment status, and economic indicators strengthen the effect estimates.

## 5. Conclusions

Across two Italian birth cohorts, maternal dietary patterns were socially stratified, whereas pre-pregnancy BMI and maternal education were more consistently associated with birth outcomes than dietary-pattern adherence per se. These findings suggest that diet during pregnancy is embedded within broader social and maternal contexts rather than operating as an entirely separable determinant of perinatal risk.

From a policy standpoint, our results support interventions targeting upstream social determinants of health, alongside strategies promoting healthy weight and diet quality before pregnancy. Key priorities include accessible preconception services embedded within primary care, targeted support for younger and socioeconomically disadvantaged women, and the integration of community-based initiatives aimed at increasing awareness of healthy behaviors during the peri-conceptional period.

In clinical practice, pattern-based dietary profiling, combined with routine assessment of pre-pregnancy BMI and key sociodemographic factors, may help identify women at higher risk and inform more precise, individualized intervention strategies, such as tailored nutritional counseling, structured weight-management pathways, and timely screening within standard maternity care. Future research should refine exposure assessment through trimester-specific dietary measures, incorporate objective nutritional biomarkers, leverage validated digital tools, and promote harmonization of instruments and protocols across cohorts to enhance comparability and accelerate translation into population-level practice.

Building on this research agenda and the DOHaD framework, the next step will be to evaluate DNA methylation profiles in a subset of children from both cohorts and to investigate whether maternal diet during pregnancy is associated with epigenetic programming, potentially providing a biological mechanism linking early-life exposures to later adverse child outcomes.

## Figures and Tables

**Table 1:** Variable coding and definitions for the Piccolipiù and MAMI-MED cohorts.

Age at Delivery	Continuous Variable	
**Gestational age (weeks)**	Continuous variable	
**Pre-pregnancy BMI**	Weight in kilograms divided by height in squared meters.	
**Nutritional status**	According to World Health Organization (WHO) criteria: underweight (<18.5); normal weight (18.5–24.9); overweight (25–29.9); obese (≥30)	
**Smoking during pregnancy**	Yes/no	
**Parity**	Nulliparous: She has never given birth; Uniparous or multiparous: She has given birth at least once.	
**Preterm birth**	Yes: delivery before 37 completed weeks of gestation	
**Type of delivery**	Natural/cesarean	
**Birth weight**	Continuous variable	
**Birth length**	Continuous variable	
**Macrosomia**	Yes: ≥4000 g	
**SGA/AGA/LGA**	Weight-for-gestational-age was categorized as small for gestational age (SGA), appropriate for gestational age (AGA), or large for gestational age (LGA) according to sex- and gestational age-specific national reference percentiles: SGA < 10th percentile, AGA 10–90th percentile, and LGA > 90th percentile.	
**LBW, low birth weight**	Yes: ≤2500 g	
**Sex**	Male/female	
**Variables with different coding in the Piccolipiù (left) and MAMI-MED (right) cohorts**		
**Parental employment**	Always worked: employed both before and after childbirth not continuous: employed only before or after childbirth	Employed: both full-time and part-time unemployed: including students and housewives
**Maternal and Paternal education at childbirth**	Low: no education, primary school, lower secondary school Medium: secondary high school high: post-secondary education or university degree	Low: ≤8 years of schooling medium: 9–13 years high: >13 years
**Smoking before pregnancy**	Yes/no	Current smoker/Not smoker
**Alcohol intake during pregnancy**	Yes/no	Assessed using the food frequency questionnaire
**Center of recruitment**	Turin, Trieste, Florence, Viareggio, Rome	Catania
**Variables with Cohort-Specific Coding in the Piccolipiù (left) and MAMI-MED (right) cohorts**		
**Equivalized household income** **indicator (EHII)**	The country-specific cut-off corresponding to the upper tertile of the 2011 EU-SILC reference distribution (households with at least one child ≤16 years) was EUR 1572.6, corresponding to a log-income value of 7.36. Participants with predicted log-EHII values <7.36 were classified as low/medium-income, and those with values ≥7.36 as high-income.	Na

**Table 2:** Maternal characteristics and socio-demographic characteristics by category of adherence to dietary patterns in the Piccolipiù cohort.

Adherence to Dietary Pattern						
Characteristic	Exclusively Western (n = 343)	Preferably Western (n = 735)	No Preference (n = 1087)	Preferably Prudent (n = 715)	Exclusively Prudent (n = 354)	*p*-Value
Age, years, median (IQR^d^)	31 (7)	33 (7)	34 (6)	35 (6)	35.5 (6)	<0.001
Weight, kg, median (IQR^d^)	60 (14)	60 (13)	60 (13)	59 (12)	59 (11)	0.319
Pre-Gestational BMI, kg/m^2^, median (IQR^d^)	22 (4.6)	21.5 (4)	21.8 (4.4)	21.5 (4.1)	21.3 (4)	0.011
Nutritional status						0.126
Underweight	7.85%	7.89%	6.8%	7.97%	8.76%	
Normal weight	66.57%	71.16%	71.51%	73.29%	74.01%	
Overweight	15.7%	13.33%	15.72%	13.43%	12.15%	
Obesity	8.72%	6.94%	4.96%	4.9%	4.52%	
EHII, Median (IQR^d^)	1592.11 (630.72)	1695.98 (661.52)	1743.68 (657.59)	1859.67 (586.18)	2002.76 (584.43)	<0.001
EHII, (%)						<0.001
Low/Medium (≤1572.6)	57.27%	49.25%	43.01%	36.22%	27.12%	
High (>1572.6)	34.01%	44.22%	50.74%	56.92%	67.23%	
Employment status						<0.001
Continuous	22.38%	34.29%	35.85%	45.45%	44.07%	
Not continuous	77.33%	65.71%	64.06%	54.55%	55.93%	
Education level						<0.001
Low	24.13%	12.79%	12.13%	6.57%	4.8%	
Medium	46.51%	49.25%	43.01%	40.42%	34.75%	
High	29.07%	37.96%	44.76%	53.01%	60.45%	
Center, (%)						0.019
Central Italy (FI, LU, RM)	61.92%	60.82%	66.36%	67.97%	67.23%	
Northern Italy (TO, TS)	38.08%	39.18%	33.55%	32.03%	32.77%	

Continuous variables are presented as median and (interquartile range, IQR), while categorical variables are shown as percentage of frequency. The Kruskal–Wallis test was used for continuous variables due to their non-normal distribution, and the chi-square test was used for categorical variables. BMI: body mass index.

**Table 3:** Maternal characteristics and socio-demographic determinants by category of adherence to dietary patterns in the MAMI-MED cohort.

Adherence to Dietary Pattern						
Characteristic	Exclusively Western (n = 215)	Preferably Western (n = 374)	No Preference (n = 402)	Preferably Prudent (n = 356)	Exclusively Prudent (n = 217)	*p*-Value
Age, years, median (IQR)	28.0 (8)	30.0 (7)	31.0 (7)	32.0 (5)	32.0 (5)	<0.001
Weight, kg, median (IQR)	62.0 (19.0)	64.0 (16.0)	63.0 (16.0)	64.0 (17.0)	63.0 (18.3)	0.545
Pre-Gestational BMI, kg/m^2^, median (IQR)	22.9 (6.6)	23.6 (6.3)	23.3 (5.4)	23.3 (6.3)	22.9 (5.6)	0.292
Nutritional status						0.683
Underweight	8.4%	4.0%	6.0%	6.2%	6.9%	
Normal weight	57.2%	57.1%	60.5%	56.9%	59.9%	
Overweight	19.5%	25.3%	20.8%	23.7%	21.2%	
Obese	14.9%	13.5%	12.8%	13.2%	12.0%	
Employment status						
Employed	32.4%	46.6%	54.4%	60.0%	65.1%	<0.001
Not Employed	67.6%	53.4%	45.6%	40.0%	34.9%	
Educational level						
Low	45.1%	31.4%	22.1%	16.7%	8.4%	<0.001
Medium	48.4%	49.3%	51.1%	54.4%	49.1%	
High	6.6%	19.3%	26.8%	28.9%	42.5%	
Occupational status						
Full-time	17.8%	25.6%	29.6%	39.7%	41.4%	<0.001
Part-time	14.6%	20.9%	24.8%	20.3%	23.7%	
Economically inactive	67.6%	57.1%	45.7%	40.0%	34.9%	

Variables are reported as median and (interquartile range difference, IQR) or percentage. DP, dietary pattern; BMI, body mass index. The Kruskal–Wallis test was used to analyze continuous variables due to the non-normal distribution of data.

## Data Availability

The data presented in this study are available on reasonable request from the corresponding author due to privacy constraints. All proposals requesting data access will need to specify how the data will be used.

## Supplementary Materials

The following supporting information can be downloaded at: https://www.mdpi.com/article/10.3390/nu18071065/s1, Table S1. Characteristics of women and newborns belonging to the Piccolipiù cohort; Table S2. Characteristics of women belonging to the MAMI-MED cohort; Table S3. Maternal and neonatal outcomes in the Piccolipiù cohort; Table S4. Maternal and neonatal outcomes in the MAMI-MED cohort; Table S5. Association between maternal dietary pattern adherence and preterm birth in the Piccolipiù cohort using multivariable logistic regression models; Table S6. Association between maternal dietary pattern adherence and preterm birth in the MAMI MED cohort using multivariable logistic regression models; Table S7. Association between maternal dietary pattern adherence and low birth weight in the Piccolipiù cohort using multivariable logistic regression models; Table S8. Association between maternal dietary pattern adherence and low birth weight in the MAMI-MED cohort using multivariable logistic regression models; Table S9. Association between maternal dietary pattern adherence and macrosomia in the Piccolipiù cohort using multivariable logistic regression models; Table S10. Association between maternal dietary pattern adherence and macrosomia in the MAMI MED cohort using multivariable logistic regression models; Table S11. Association between maternal dietary pattern adherence and LGA vs. AGA in the Piccolipiù cohort using multivariable logistic regression models; Table S12. Association between maternal dietary pattern adherence and LGA vs. AGA in the MAMI MED cohort using multivariable logistic regression models; Table S13. Association between maternal dietary pattern adherence and SGA vs. AGA in the Piccolipiù cohort using multivariable logistic regression models; Table S14. Association between maternal dietary pattern adherence and SGA vs. AGA in the MAMI MED cohort using multivariable logistic regression model.
