# Supplementary material for: Maternal Dietary Patterns, Socioeconomic Conditions, and Birth Outcomes in the MAMI-MED and Piccolipiù Italian Birth Cohorts
Source: Nutrients. 2026 Mar 26;18(7):1065. doi: 10.3390/nu18071065 (PMC13074797; doi:10.3390/nu18071065)
Supplement: Supplementary file 1 [file nutrients-18-01065-s001.zip › nutrients-4207500-supplementary.docx]

**Table S1**. Characteristics of women and newborns belonging to the Piccolipiù cohort.

| **Characteristics** | **Overall**  **(n=3234)** |  |
| --- | --- | --- |
| Age at delivery (years) | 34 (7) |  |
| **Maternal education at childbirth** |  |  |
| Low | 11.5% |  |
| Medium | 43.4% |  |
| High | 45.14% |  |
| NA | 0.1% |  |
| **Paternal education at childbirth** |  |  |
| Low | 21.78% |  |
| Medium | 45.0% |  |
| High | 32.3% |  |
| NA | 1.1% |  |
| **Maternal employment** |  |  |
| Always worked | 37.1% |  |
| Not continuous | 62. 9% |  |
| **Paternal employment** |  |  |
| Always worked | 46. 8% |  |
| Not continuous | 53. 2% |  |
| **Equivalized Household Income Indicator (EHII)** |  |  |
| Low/Medium | 42.7% |  |
| High | 50.7% |  |
| NA | 6.6% |  |
| **City of recruitment** |  |  |
| Florence | 16.2% |  |
| Viareggio | 15.9% |  |
| Rome | 33.0% |  |
| Turin | 16.7% |  |
| Trieste | 18.2% |  |
| **Parity** |  |  |
| Nulliparous | 58.0% |  |
| Uniparous or multiparous | 41.8% |  |
| NA | 0.2% |  |
| **Smoking before pregnancy** |  |  |
| NO | 50.8% |  |
| YES | 48.9% |  |
| NA | 0.3% |  |
| **Alcohol in pregnancy** |  |  |
| NO | 54.6% |  |
| YES | 44.6% |  |
| NA | 0.8% |  |
| Pre-Gestational BMI (kg/m^2^) | | 21.7 (4.3) |
| **Nutritional status** |  |  |
| Under weight | 7.6% |  |
| Normal weight | 72.5% |  |
| Overweight | 13.4% |  |
| Obese | 5.7% |  |
| NA | 0.7% |  |
| **Child sex** |  |  |
| Female | 48.8% |  |
| Male | 51.2% |  |

*Data ​​are expressed as median (Interquartile Range difference, IQRd) or percentage of frequency, as appropriate. *BMI: body mass index.* *NA: Not Available*

**Table S2.**  Characteristics of women belonging to the MAMI-MED cohort.

| *Characteristics* | *Overall*  *(n = 1564)* |
| --- | --- |
| Age (Years) | 31.0 (7.0) |
| Educational Level |  |
| Low | 24.3% |
| Medium | 50.8% |
| High | 24.9% |
| Employment |  |
| Yes | 52.3% |
| Not | 47.7% |
| Smoker |  |
| Yes | 9.3% |
| Not | 90.7% |
| Weight, kg | 63.0 (17.0) |
| Pre-Gestational BMI, kg/m^2^ | 23.3 (6.0) |
| Nutritional status |  |
| Under weight | 6.0% |
| Normal weight | 58.3% |
| Overweight | 22.4% |
| Obese | 13.2% |
| Gestational Weight Gain (GWG) ^a^ |  |
| Reduced | 38.2% |
| Adequate | 32.2% |
| Excessive | 29.6% |
| Having Children ^b^ |  |
| Yes | 46.4% |
| No | 53.6% |

*Data are presented as median (Interquartile Range difference, IQRd) or frequency (percentage). ^a^ Categorized based on pre-pregnancy BMI and the Institute of Medicine guidelines. ^b^ Defined as having at least one other child besides the one included in this study.

**Table S3.** Maternal and neonatal outcomes in the Piccolipiù cohort.

| Characteristics | Overall  (n=3234) |
| --- | --- |
| Gestational week at delivery (weeks) | **40 (2)** |
| Preterm birth | **4.8%** |
| NA | **0.1%** |
| Low birth weight (<2500) | **2.7%** |
| NA | **0.5%** |
| Birth weight (grams) | **3340 (580)** |
| Birth length (cm) | **50 (2)** |
| Macrosomia (birth weight>4000) | **6.4%** |
| NA | **0.5%** |
| Small-for-gestational age, SGA | **11%** |
| Adeguate-for-gestational age, AGA | **79.7%** |
| Large-for-gestational age, LGA | **8.6%** |
| NA | **0.7%** |
| Mode of delivery |  |
| Natural | **72.8%** |
| Cesarean | **27.2%** |

*Results are reported as median (Interquartile Range difference, IQRd) or percentage of frequency.

**Table S4.** Maternal and neonatal outcomes in the MAMI-MED cohort.

| Characteristics | Overall  (n= 1564) |
| --- | --- |
| Gestational age at delivery (weeks) | 39 (2) |
| Preterm birth | 6.0% |
| Low birth weight | 6.3% |
| Birth weight (grams) | 3300 (600) |
| Birth length (cm) | 50.0 (2.0) |
| Macrosomia | 3.9% |
| Small-for-gestational age | 9.6% |
| Adeguate-for-gestational age | 80.7% |
| Large-for-gestational age | 9.7% |
| Type of delivery | |
| Natural | 69.1% |
| Caesarian | 30.9%% |

^*^ Results are reported as median (Interquartile Range difference, IQRd) or frequency (%)

**Table S5.** Association between maternal dietary pattern adherence and preterm birth in the Piccolipiù cohort using multivariable logistic regression models.

|  | **Characteristics** | **OR ^a^** | **95% CI** | ***p*-Value** |
| --- | --- | --- | --- | --- |
| **Model 1** | **Adherence To Dietary Pattern** |  |  |  |
|  | Exclusively Western | 1.193 | 0.678 - 2.015 | 0.524 |
|  | Preferably Western | 0.863 | 0.539 - 1.359 | 0.530 |
|  | No Preference | Ref. |  |  |
|  | Preferably Prudent | 1.012 | 0.644 - 1.572 | 0.957 |
|  | Exclusively Prudent | 1.218 | 0.7 - 2.04 | 0.468 |
| **Model 2** | **Adherence To Dietary Pattern** |  |  |  |
|  | Exclusively Western | 1.173 | 0.663 - 1.995 | 0.568 |
|  | Preferably Western | 0.861 | 0.537 - 1.358 | 0.526 |
|  | No Preference | Ref. |  |  |
|  | Preferably Prudent | 1.023 | 0.65 - 1.591 | 0.921 |
|  | Exclusively Prudent | 1.241 | 0.712 - 2.088 | 0.429 |
|  | **Age (Years)** | 1.001 | 0.968 - 1.035 | 0.954 |
|  | **Pre-Gestational BMI** | 1.044 | 1.004 - 1.084 | **0.028** |
| **Model 3** | **Adherence To Dietary Pattern** |  |  |  |
|  | Exclusively Western | 1.017 | 0.555 - 1.78 | 0.954 |
|  | Preferably Western | 0.817 | 0.501 - 1.309 | 0.409 |
|  | No Preference | Ref. |  |  |
|  | Preferably Prudent | 0.957 | 0.589 - 1.528 | 0.855 |
|  | Exclusively Prudent | 1.238 | 0.687 - 2.14 | 0.459 |
|  | **Age (Years)** | 1.005 | 0.97 - 1.041 | 0.801 |
|  | **Pre-Gestational BMI** | 1.039 | 0.997 - 1.08 | 0.059 |
|  | **Educational Level** |  |  |  |
|  | Low | 2.923 | 1.598 - 5.288 | **<0.001** |
|  | Medium | 1.768 | 1.169 - 2.687 | **0.007** |
|  | High | Ref. |  |  |
|  | **Occupational Status** |  |  |  |
|  | Always Worked | Ref. |  |  |
|  | Not Continuous | 1.279 | 0.761 - 2.089 | 0.337 |
|  | **EHII** |  |  |  |
|  | Low/Medium | 0.675 | 0.432 - 1.046 | 0.081 |
|  | High | Ref. |  |  |

^a^ Results are expressed as odds ratios (OR) with 95% confidence intervals (CI) and corresponding *p*-values. “Ref” indicates the reference category used for each categorical variable in the model. The dependent variable in the logistic regression model was preterm birth (PTB) status, defined as whether or not the birth was preterm.

**Table S6.** Association between maternal dietary pattern adherence and preterm birth in the MAMI MED cohort using multivariable logistic regression models.

|  | **Characteristics** | **OR ^a^** | **95% CI** | ***p*-Value** |
| --- | --- | --- | --- | --- |
| **Model 1** | **Adherence to Dietary pattern** |  |  |  |
|  | Exclusively Western | 0.735 | 0.346–1.561 | 0.423 |
|  | Preferably Western | 0.986 | 0.549–1.769 | 0.962 |
|  | Preferably prudent | 0.943 | 0.518–1.716 | 0.848 |
|  | Exclusively prudent | 1.034 | 0.526–2.034 | 0.922 |
|  | No preference | Ref. |  |  |
| **Model 2** | **Adherence to Dietary pattern** |  |  |  |
|  | Exclusively Western | 0.709 | 0.335–1.535 | 0.393 |
|  | Preferably Western | 0.961 | 0.529–1.716 | 0.872 |
|  | Preferably prudent | 0.929 | 0.509–1.702 | 0.817 |
|  | Exclusively prudent | 1.048 | 0.528–2.071 | 0.898 |
|  | No preference | Ref. |  |  |
|  | **Age (years)** | 0.996 | 0.951–1.040 | 0.808 |
|  | **Pre-pregnancy BMI** | 1.045 | 1.009–1.082 | **0.013** |
| **Model 3** | **Adherence to Dietary pattern** |  |  |  |
|  | Exclusively Western | 0.647 | 0.299–1.401 | 0.270 |
|  | Preferably Western | 0.978 | 0.539-1.777 | 0.942 |
|  | Preferably prudent | 0.934 | 0.503–1.732 | 0.828 |
|  | Exclusively prudent | 1.234 | 0.616–2.473 | 0.552 |
|  | No preference | Ref. |  |  |
|  | **Age (years)** | 1.021 | 0.975–1.069 | 0.371 |
|  | **Pre-pregnancy BMI** | 1.031 | 0.994–1.069 | 0.097 |
|  | **Educational level** |  |  |  |
|  | Low | 2.585 | 1.166-5.729 | **0.019** |
|  | Medium | 2.112 | 1.075-4.149 | **0.030** |
|  | High | Ref. |  |  |
|  | **Occupational Status** |  |  |  |
|  | Unemployed | 1.187 | 0.735-1.917 | 0.482 |
|  | Employed | Ref |  |  |

^a^ Results are expressed as odds ratios (OR) with 95% confidence intervals (CI) and corresponding *p*-values. “Ref” indicates the reference category used for each categorical variable in the model. The dependent variable in the logistic regression model was preterm birth (PTB) status, defined as whether or not the birth was preterm.

**Table S7.** Association between maternal dietary pattern adherence and low birth weight in the Piccolipiù cohort using multivariable logistic regression models.

|  | **Characteristics** | **OR ^a^** | **95% CI** | ***p*-Value** |
| --- | --- | --- | --- | --- |
| **Model 1** | **Adherence to Dietary pattern** |  |  |  |
|  | Exclusively Western | 0.805 | 0.376 - 1.567 | 0.546 |
|  | Preferably Western | 0.711 | 0.399 - 1.224 | 0.230 |
|  | No preference | Ref. |  |  |
|  | Preferably Prudent | 0.498 | 0.254 - 0.914 | **0.031** |
|  | Exclusively Prudent | 0.544 | 0.221 - 1.153 | 0.142 |
| **Model 2** | **Adherence to Dietary pattern** |  |  |  |
|  | Exclusively Western | 0.798 | 0.37 - 1.568 | 0.535 |
|  | Preferably Western | 0.707 | 0.396 - 1.218 | 0.223 |
|  | No preference | Ref. |  |  |
|  | Preferably Prudent | 0.496 | 0.253 - 0.912 | **0.031** |
|  | Exclusively Prudent | 0.544 | 0.221 - 1.159 | 0.145 |
|  | **Age (years)** | 0.996 | 0.954 - 1.041 | 0.871 |
|  | **Pre-Gestational BMI** | 0.999 | 0.943 - 1.053 | 0.977 |
| **Model 3** | **Adherence to Dietary pattern** |  |  |  |
|  | Exclusively Western | 0.634 | 0.269 - 1.323 | 0.256 |
|  | Preferably Western | 0.680 | 0.375 - 1.189 | 0.188 |
|  | No preference | Ref. |  |  |
|  | Preferably Prudent | 0.492 | 0.244 - 0.923 | **0.035** |
|  | Exclusively Prudent | 0.587 | 0.237 - 1.261 | 0.206 |
|  | **Age (years)** | 1.016 | 0.971 - 1.064 | 0.488 |
|  | **Pre-Gestational BMI** | 0.996 | 0.939 - 1.051 | 0.884 |
|  | **Educational level** |  |  |  |
|  | Low | 2.306 | 1.048 - 4.936 | **0.034** |
|  | Medium | 1.670 | 0.982 - 2.863 | 0.059 |
|  | High | Ref. |  |  |
|  | **Occupational Status** |  |  |  |
|  | Always worked | Ref. |  |  |
|  | Not continuous | 1.248 | 0.636 - 2.326 | 0.499 |
|  | **EHII** |  |  |  |
|  | Low/Medium | 0.810 | 0.46 - 1.413 | 0.461 |
|  | High | Ref. |  |  |

^a^ Results are expressed as odds ratios (OR) with 95% confidence intervals (CI) and corresponding *p*-values. “Ref” indicates the reference category used for each categorical variable in the model. The dependent variable in the logistic regression model was LBW (yes/ not).

**Table S8.** Association between maternal dietary pattern adherence and low birth weight in the MAMI-MED cohort using multivariable logistic regression models.

|  | **Characteristics** | **OR ^a^** | **95% CI** | ***p*-Value** |
| --- | --- | --- | --- | --- |
| **Model 1** | **Adherence to Dietary pattern** |  |  |  |
|  | Exclusively Western | 1.024 | 0.510–2.054 | 0.947 |
|  | Preferably Western | 1.080 | 0.602–1.937 | 0.796 |
|  | Preferably prudent | 0.888 | 0.478–1.650 | 0.707 |
|  | Exclusively prudent | 1.425 | 0.755–2.688 | 0.275 |
|  | No preference | Ref. |  |  |
| **Model 2** | **Adherence to Dietary pattern** |  |  |  |
|  | Exclusively Western | 1.091 | 0.540–2.203 | 0.809 |
|  | Preferably Western | 1.098 | 0.611–1.973 | 0.754 |
|  | Preferably prudent | 0.833 | 0.447–1.554 | 0.566 |
|  | Exclusively prudent | 1.318 | 0.694–2.503 | 0.399 |
|  | No preference | Ref. |  |  |
|  | **Age (years)** | 1.043 | 0.997–1.090 | 0.066 |
|  | **Pre-pregnancy BMI** | 1.027 | 0.991–1.064 | 0.148 |
| **Model 3** | **Adherence to Dietary pattern** |  |  |  |
|  | Exclusively Western | 0.825 | 0.397–1.715 | 0.606 |
|  | Preferably Western | 1.058 | 0.586–1.911 | 0.851 |
|  | Preferably prudent | 0.864 | 0.462–1.617 | 0.648 |
|  | Exclusively prudent | 1.525 | 0.795–2.923 | 0.204 |
|  | No preference | Ref. |  |  |
|  | **Age (years)** | 1.065 | 1.018–1.114 | **0.006** |
|  | **Pre-pregnancy BMI** | 1.016 | 0.980–1.054 | 0.381 |
|  | **Educational level** |  |  |  |
|  | Low | 2.074 | 1.007-4.273 | **0.048** |
|  | Medium | 1.462 | 0.797-2.682 | 0.219 |
|  | High | Ref. |  |  |
|  | **Occupational Status** |  |  |  |
|  | Unemployed | 1.539 | 0.954-2.483 | 0.077 |
|  | Employed | Ref. |  |  |

^a^ Results are expressed as odds ratios (OR) with 95% confidence intervals (CI) and corresponding *p*-values. “Ref” indicates the reference category used for each categorical variable in the model. The dependent variable in the logistic regression model was LBW (yes/ not).

**Table S9.** Association between maternal dietary pattern adherence and macrosomia in the Piccolipiù cohort using multivariable logistic regression models.

|  | **Characteristics** | **OR ^a^** | **95% CI** | ***p*-Value** |
| --- | --- | --- | --- | --- |
| **Model 1** | **Adherence to Dietary pattern** |  |  |  |
|  | Exclusively Western | 1.111 | 0.659 - 1.806 | 0.681 |
|  | Preferably Western | 0.982 | 0.653 - 1.463 | 0.930 |
|  | No preference | Ref. |  |  |
|  | Preferably Prudent | 1.462 | 1.01 - 2.114 | **0.043** |
|  | Exclusively Prudent | 0.976 | 0.568 - 1.61 | 0.928 |
| **Model 2** | **Adherence to Dietary pattern** |  |  |  |
|  | Exclusively Western | 1.084 | 0.639 - 1.776 | 0.756 |
|  | Preferably Western | 0.989 | 0.655 - 1.478 | 0.956 |
|  | No preference | Ref. |  |  |
|  | Preferably Prudent | 1.523 | 1.048 - 2.21 | **0.027** |
|  | Exclusively Prudent | 1.032 | 0.598 - 1.711 | 0.905 |
|  | **Age (years)** | 0.996 | 0.968 - 1.026 | 0.812 |
|  | **Pre-Gestational BMI** | 1.071 | 1.036 - 1.105 | **<0.001** |
| **Model 3** | **Adherence to Dietary pattern** |  |  |  |
|  | Exclusively Western | 1.168 | 0.676 - 1.948 | 0.563 |
|  | Preferably Western | 0.972 | 0.633 - 1.478 | 0.896 |
|  | No preference | Ref. |  |  |
|  | Preferably Prudent | 1.558 | 1.058 - 2.295 | **0.024** |
|  | Exclusively Prudent | 1.061 | 0.603 - 1.792 | 0.831 |
|  | **Age (years)** | 0.998 | 0.968 - 1.03 | 0.924 |
|  | **Pre-Gestational BMI** | 1.066 | 1.029 - 1.103 | **<0.001** |
|  | **Educational level** |  |  |  |
|  | Low | 0.528 | 0.282 - 0.944 | **0.037** |
|  | Medium | 0.850 | 0.6 - 1.202 | 0.358 |
|  | High | Ref. |  |  |
|  | **Occupational Status** |  |  |  |
|  | Always worked | Ref. |  |  |
|  | Not continuous | 1.399 | 0.921 - 2.093 | 0.108 |
|  | **EHII** |  |  |  |
|  | Low/Medium | 1.354 | 0.931 - 1.964 | 0.111 |
|  | High | Ref. |  |  |

^a^ Results are expressed as odds ratios (OR) with 95% confidence intervals (CI) and corresponding *p*-values. “Ref” indicates the reference category used for each categorical variable in the model. The dependent variable in the logistic regression model was macrosomia (yes/ not).

**Table S10.** Association between maternal dietary pattern adherence and macrosomia in the MAMI MED cohort using multivariable logistic regression models.

|  | **Characteristics** | **OR ^a^** | **95% CI** | ***p*-Value** |
| --- | --- | --- | --- | --- |
| **Model 1** | **Adherence to Dietary pattern** |  |  |  |
|  | Exclusively Western | 0.946 | 0.417–2.143 | 0.894 |
|  | Preferably Western | 0.709 | 0.337–1.493 | 0.366 |
|  | Preferably Prudent | 0.876 | 0.429–1.788 | 0.716 |
|  | Exclusively Prudent | 0.718 | 0.295–1.747 | 0.465 |
|  | No preference | Ref. |  |  |
| **Model 2** | **Adherence to Dietary pattern** |  |  |  |
|  | Exclusively Western | 0.917 | 0.397–2.118 | 0.840 |
|  | Preferably Western | 0.711 | 0.333–1.518 | 0.379 |
|  | Preferably Prudent | 0.949 | 0.458–1.964 | 0.888 |
|  | Exclusively Prudent | 0.818 | 0.331–2.022 | 0.664 |
|  | No preference | Ref. |  |  |
|  | **Age (years)** | 0.969 | 0.917–1.023 | 0.252 |
|  | **Pre-pregnancy BMI** | 1.054 | 1.012–1.098 | **0.012** |
| **Model 3** | **Adherence to Dietary pattern** |  |  |  |
|  | Exclusively Western | 0.951 | 0.406–2.229 | 0.908 |
|  | Preferably Western | 0.733 | 0.342–1.569 | 0.423 |
|  | Preferably prudent | 0.952 | 0.460–1.970 | 0.894 |
|  | Exclusively prudent | 0.814 | 0.328–2.020 | 0.656 |
|  | No preference | Ref. |  |  |
|  | **Age (years)** | 0.966 | 0.911–1.023 | 0.235 |
|  | **Pre-pregnancy BMI** | 1.057 | 1.014–1.103 | **0.010** |
|  | **Educational level** |  |  |  |
|  | Low | 1.014 | 0.410-2.507 | 0.976 |
|  | Medium | 0.991 | 0.487-2.018 | 0.980 |
|  | High | Ref. |  |  |
|  | **Occupational Status** |  |  |  |
|  | Unemployed | 0.812 | 0.448-1.472 | 0.492 |
|  | Employed | Ref |  |  |

^a^ Results are expressed as odds ratios (OR) with 95% confidence intervals (CI) and corresponding *p*-values. “Ref” indicates the reference category used for each categorical variable in the model. The dependent variable in the logistic regression model was macrosomia (yes/ not).

**Table S11.** Association between maternal dietary pattern adherence and LGA vs AGA in the Piccolipiù cohort using multivariable logistic regression models.

|  | **Characteristics** | **OR ^a^** | **95% CI** | ***p*-Value** |
| --- | --- | --- | --- | --- |
| **Model 1** | **Adherence to Dietary pattern** |  |  |  |
|  | Exclusively Western | 1.142 | 0.721 - 1.762 | 0.559 |
|  | Preferably Western | 1.136 | 0.801 - 1.604 | 0.472 |
|  | No preference | Ref. |  |  |
|  | Preferably Prudent | 1.457 | 1.043 - 2.032 | **0.027** |
|  | Exclusively Prudent | 1.182 | 0.755 - 1.808 | 0.452 |
| **Model 2** | **Adherence to Dietary pattern** |  |  |  |
|  | Exclusively Western | 1.115 | 0.7 - 1.732 | 0.636 |
|  | Preferably Western | 1.143 | 0.803 - 1.619 | 0.455 |
|  | No preference | Ref. |  |  |
|  | Preferably Prudent | 1.489 | 1.064 - 2.082 | **0.020** |
|  | Exclusively Prudent | 1.177 | 0.745 - 1.816 | 0.471 |
|  | **Age (years)** | 1.001 | 0.975 - 1.027 | 0.948 |
|  | **Pre-Gestational BMI** | 1.073 | 1.043 - 1.104 | **<0.001** |
| **Model 3** | **Adherence to Dietary pattern** |  |  |  |
|  | Exclusively Western | 1.112 | 0.684 - 1.758 | 0.659 |
|  | Preferably Western | 1.102 | 0.765 - 1.58 | 0.600 |
|  | No preference | Ref. |  |  |
|  | Preferably Prudent | 1.563 | 1.106 - 2.208 | **0.011** |
|  | Exclusively Prudent | 1.180 | 0.733 - 1.851 | 0.482 |
|  | **Age (years)** | 1.006 | 0.979 - 1.034 | 0.661 |
|  | **Pre-Gestational BMI** | 1.069 | 1.037 - 1.101 | **<0.001** |
|  | **Educational level** |  |  |  |
|  | Low | 0.778 | 0.466 - 1.271 | 0.326 |
|  | Medium | 0.925 | 0.681 - 1.255 | 0.616 |
|  | High | Ref. |  |  |
|  | **Occupational Status** |  |  |  |
|  | Always worked | Ref. |  |  |
|  | Not continuous | 1.393 | 0.965 - 1.991 | 0.072 |
|  | **EHII** |  |  |  |
|  | Low/Medium | 1.262 | 0.907 - 1.751 | 0.165 |
|  | High | Ref. |  |  |

^a^ Results are expressed as odds ratios (OR) with 95% confidence intervals (CI) and corresponding *p*-values. “Ref” indicates the reference category used for each categorical variable in the model. The dependent variable in the logistic regression model was LGA vs AGA.

**Table S12.** Association between maternal dietary pattern adherence and LGA vs AGA in the MAMI MED cohort using multivariable logistic regression models.

|  | **Characteristics** | **OR ^a^** | **95% CI** | ***p*-value** |
| --- | --- | --- | --- | --- |
| **Model 1** | **Adherence to Dietary pattern** |  |  |  |
|  | Exclusively Western | 1.223 | 0.723–2.066 | 0.453 |
|  | Preferably Western | 0.755 | 0.462–1.232 | 0.260 |
|  | Preferably Prudent | 0.847 | 0.523–1.373 | 0.501 |
|  | Exclusively Prudent | 0.801 | 0.447–1.435 | 0.456 |
|  | No preference | Ref. |  |  |
| **Model 2** | **Adherence to Dietary pattern** |  |  |  |
|  | Exclusively Western | 1.176 | 0.688–2.011 | 0.553 |
|  | Preferably Western | 0.731 | 0.445–1.201 | 0.216 |
|  | Preferably Prudent | 0.863 | 0.529–1.409 | 0.557 |
|  | Exclusively Prudent | 0.846 | 0.468-1.530 | 0.581 |
|  | No preference | Ref. |  |  |
|  | **Age (years)** | 0.983 | 0.949–1.019 | 0.361 |
|  | **Pre-pregnancy BMI** | 1.058 | 1.028-1.089 | **< 0.001** |
| **Model 3** | **Adherence to Dietary pattern** |  |  |  |
|  | Exclusively Western | 1.205 | 0.697–2.081 | 0.504 |
|  | Preferably Western | 0.769 | 0.466–1.270 | 0.305 |
|  | Preferably Prudent | 0.893 | 0.546–1.462 | 0.653 |
|  | Exclusively Prudent | 0.885 | 0.486–1.611 | 0.690 |
|  | No preference | Ref. |  |  |
|  | **Age (years)** | 0.989 | 0.952–1.027 | 0.568 |
|  | **Pre-pregnancy BMI** | 1.059 | 1.028-1.091 | **< 0.001** |
|  | **Educational level** |  |  |  |
|  | Low | 1.122 | 0.614-2.051 | 0.708 |
|  | Medium | 1.097 | 0.683-1.764 | 0.702 |
|  | High | Ref. |  |  |
|  | **Occupational Status** |  |  |  |
|  | Unemployed | 0.951 | 0.639-1.415 | 0.806 |
|  | Employed | Ref |  |  |

^a^ Results are expressed as odds ratios (OR) with 95% confidence intervals (CI) and corresponding *p*-values. “Ref” indicates the reference category used for each categorical variable in the model. The dependent variable in the logistic regression model was LGA vs AGA.

**Table S13.** Association between maternal dietary pattern adherence and SGA vs AGA in the Piccolipiù cohort using multivariable logistic regression models.

|  | **Characteristics** | **OR ^a^** | **95% CI** | ***p*-Value** |
| --- | --- | --- | --- | --- |
| **Model 1** | **Adherence to Dietary pattern** |  |  |  |
|  | Exclusively Western | 0.992 | 0.662 - 1.455 | 0.966 |
|  | Preferably Western | 0.973 | 0.716 - 1.316 | 0.858 |
|  | No preference | Ref. |  |  |
|  | Preferably Prudent | 1.166 | 0.865 - 1.566 | 0.310 |
|  | Exclusively Prudent | 0.934 | 0.621 - 1.375 | 0.736 |
| **Model 2** | **Adherence to Dietary pattern** |  |  |  |
|  | Exclusively Western | 1.034 | 0.687 - 1.526 | 0.868 |
|  | Preferably Western | 0.990 | 0.727 - 1.342 | 0.951 |
|  | No preference | Ref. |  |  |
|  | Preferably Prudent | 1.133 | 0.839 - 1.525 | 0.412 |
|  | Exclusively Prudent | 0.909 | 0.603 - 1.341 | 0.639 |
|  | **Age (years)** | 1.009 | 0.986 - 1.033 | 0.459 |
|  | **Pre-Gestational BMI** | 0.963 | 0.932 - 0.994 | **0.022** |
| **Model 3** | **Adherence to Dietary pattern** |  |  |  |
|  | Exclusively Western | 0.947 | 0.616 - 1.424 | 0.799 |
|  | Preferably Western | 0.937 | 0.679 - 1.285 | 0.687 |
|  | No preference | Ref. |  |  |
|  | Preferably Prudent | 1.208 | 0.886 - 1.643 | 0.229 |
|  | Exclusively Prudent | 0.939 | 0.611 - 1.412 | 0.769 |
|  | **Age (years)** | 1.017 | 0.993 - 1.043 | 0.173 |
|  | **Pre-Gestational BMI** | 0.958 | 0.926 - 0.989 | **0.010** |
|  | **Educational level** |  |  |  |
|  | Low | 1.881 | 1.237 - 2.839 | **0.003** |
|  | Medium | 1.281 | 0.969 - 1.693 | 0.082 |
|  | High | Ref. |  |  |
|  | **Occupational Status** |  |  |  |
|  | Always worked | Ref. |  |  |
|  | Not continuous | 0.712 | 0.48 - 1.032 | 0.081 |
|  | **EHII** |  |  |  |
|  | Low/Medium | 1.184 | 0.884 - 1.583 | 0.256 |
|  | High | Ref. |  |  |

^a^ Results are expressed as odds ratios (OR) with 95% confidence intervals (CI) and corresponding *p*-values. “Ref” indicates the reference category used for each categorical variable in the model. The dependent variable in the logistic regression model was SGA vs AGA.

**Table S14.** Association between maternal dietary pattern adherence and SGA vs AGA in the MAMI MED cohort using multivariable logistic regression model.

|  | **Characteristics** | **OR ^a^** | **95% CI** | ***p*-value** |
| --- | --- | --- | --- | --- |
| **Model 1** | **Adherence to Dietary pattern** |  |  |  |
|  | Exclusively Western | 1.266 | 0.740–2.166 | *p* = 0.389 |
|  | Preferably Western | 0.760 | 0.459–1.261 | *p* = 0.288 |
|  | Preferably Prudent | 0.747 | 0.446–1.251 | *p* = 0.267 |
|  | Exclusively Prudent | 1.342 | 0.798–2.258 | *p* = 0.267 |
|  | No preference | Ref. |  |  |
| **Model 2** | **Adherence to Dietary pattern** |  |  |  |
|  | Exclusively Western | 1.201 | 0.696–2.072 | *p* = 0.511 |
|  | Preferably Western | 0.753 | 0.453–1.251 | *p* = 0.273 |
|  | Preferably Prudent | 0.759 | 0.452–1.274 | *p* = 0.296 |
|  | Exclusively Prudent | 1.385 | 0.819–2.344 | *p* = 0.225 |
|  | No preference | Ref. |  |  |
|  | **Age (years)** | 0.981 | 0.947–1.019 | *p* = 0.337 |
|  | **Pre-pregnancy BMI** | 1.006 | 0.974–1.039 | *p* = 0.730 |
| **Model 3** | **Adherence to Dietary pattern** |  |  |  |
|  | Exclusively Western | 1.035 | 0.588–1.821 | *p* = 0.906 |
|  | Preferably Western | 0.751 | 0.451–1.250 | *p* = 0.270 |
|  | Preferably Prudent | 0.766 | 0.456–1.287 | *p* = 0.313 |
|  | Exclusively Prudent | 1.468 | 0.863–2.495 | *p* = 0.156 |
|  | No preference | Ref. |  |  |
|  | **Age (years)** | 0.991 | 0.954–1.030 | *p* = 0.659 |
|  | **Pre-pregnancy BMI** | 1.003 | 0.970-1.037 | *p* = 0.862 |
|  | **Educational level** |  |  |  |
|  | Low | 1.319 | 0.712-2.445 | *p* = 0.379 |
|  | Medium | 1.472 | 0.907-2.389 | *p* = 0.118 |
|  | High | Ref. |  |  |
|  | **Occupational Status** |  |  |  |
|  | Unemployed | 1.187 | 0.803-1.753 | *p =* 0.390 |
|  | Employed | Ref |  |  |

^a^ Results are expressed as odds ratios (OR) with 95% confidence intervals (CI) and corresponding *p*-values. “Ref” indicates the reference category used for each categorical variable in the model. The dependent variable in the logistic regression model was SGA vs AGA.
